# Associations Between Anteroposterior Occlusal Class, Musculoskeletal Pain Patterns, and Temporomandibular Disorders in Young Adults: A Cross-Sectional Study

**DOI:** 10.3390/jcm14238606

**Published:** 2025-12-04

**Authors:** Monika Nowak, Joanna Golec, Jędrzej Golec, Aneta Wieczorek

**Affiliations:** 1 Department of Kinesiotherapy and Manual Therapy, Collegium Medicum, Andrzej Frycz Modrzewski Krakow University, 30-705 Krakow, Poland; 2 Institute of Clinical Rehabilitation, University School of Physical Education in Krakow, 31-571 Krakow, Poland; joanna.golec@awf.krakow.pl; 3 Faculty of Medicine, Collegium Medicum, Andrzej Frycz Modrzewski Krakow University, 30-705 Krakow, Poland; jedrzej.golec@gmail.com; 4 Department of Prosthodontics and Orthodontics, Faculty of Medicine, Jagiellonian University Medical College, 31-007 Krakow, Poland; aneta.wieczorek@uj.edu.pl

**Keywords:** malocclusion, temporomandibular disorders, craniofacial pain, cervical spine pain, pain intensity

## Abstract

**Background:** The relationship between sagittal malocclusion, temporomandibular disorders (TMD), and musculoskeletal pain remains uncertain. **Methods:** Cross-sectional study (April 2020–August 2021) in Małopolska, Poland. Ninety participants (ages 19–35) were classified into Angle Classes I–III (n = 30 each) and examined using RDC/TMD (Axis I/II). A proprietary, nonvalidated, piloted whole-body pain-map questionnaire, presented in anterior and posterior views and subdividing the body into predefined craniofacial, spinal, and limb regions, was used to capture pain presence, Numerical Rating Scale (NRS, 0–10) scores by region, and the total number of painful sites. Group differences were analyzed using χ^2^ and Kruskal–Wallis tests with corresponding effect sizes (measures of association strength). For NRS outcomes, a minimal clinically important difference (MCID)—defined as the smallest difference in NRS considered clinically relevant—was prespecified as approximately 1 point. **Results:** Occlusal class was not associated with TMD Axis I prevalence. However, sagittal malocclusion—particularly Class III—was linked to a less favorable pain profile. Left temporal pain was more frequent in Class III than in Classes I–II (*p* = 0.024, Cramér’s V = 0.31, medium effect), and cervical spine pain occurred more often in malocclusion groups than in Class I (*p* = 0.043, Cramér’s V = 0.26, small effect), indicating statistically significant associations. Cervical pain intensity was higher in Classes II–III than in Class I, with a pooled mean difference—defined as the difference in mean NRS between the combined Classes II–III and Class I—of 1.23 NRS points (95% CI 0.38–2.08), exceeding the ≈1-point MCID and suggesting a clinically important burden. The total number of painful sites was also greater in Class III than in Class I (*p* = 0.023, η^2^ = 0.09; Δ = 1.40 sites, 95% CI 0.39–2.41), which indicates a statistically significant association with a medium effect size and a higher overall pain burden. **Conclusions**: Sagittal occlusal class was not associated with TMD diagnosis, but malocclusion—especially Class III—was associated with a more unfavorable craniofacial pain pattern and higher cervical pain burden (*p* ≤ 0.05), with effects of potential clinical relevance.

## 1. Introduction

Modern medicine increasingly emphasizes an interdisciplinary approach to the diagnosis and treatment of complex disorders. Building on this paradigm, structured care pathways for complex craniofacial conditions explicitly link dentistry with physiotherapy, neurology, otolaryngology (ENT), sleep medicine, and pain psychology [1]. In parallel, orofacial pain has been established as a formal dental specialty, thereby consolidating team-based care and shared standards across these disciplines [2]. This integration is grounded in a biopsychosocial perspective, which views pain and dysfunction as emerging from interacting biological, psychological, and social determinants and therefore supports conservative, multimodal treatment plans delivered by interdisciplinary teams. This biopsychosocial framework is also embedded in contemporary diagnostic criteria, including the DC/TMD, which integrates physical and psychosocial assessment to guide coordinated, conservative care [3]. Abnormalities of the stomatognathic system—including malocclusions and temporomandibular joint (TMJ) dysfunctions—are common health concerns in contemporary society [4,5]. An expanding body of literature highlights the potential coexistence of these conditions and their consequences, not only for the head and neck region but also for more distal body structures [6,7,8].

Malocclusions rank among the most prevalent dentofacial conditions in the general population. A recent systematic review and meta-analysis reports continent-specific estimates—Africa 81%, Europe 72%, the Americas 53%, and Asia 48%—with a pooled global prevalence of approximately 54% [4]. Longitudinal cohort studies indicate that malocclusion severity tends to worsen from adolescence into mid-adulthood, with measurable changes in occlusal traits between ages 15 and 45 years [9,10]. Beyond their high prevalence, malocclusions may compromise fundamental physiological functions such as respiration, mastication, and biting. Reported sequelae also include speech disturbances, snoring, obstructive sleep apnea, dental trauma, periodontal disease, and degenerative changes in the TMJ. In addition to these functional and systemic effects, malocclusions exert a significant psychosocial burden by impairing facial and dental esthetics, thereby negatively influencing self-esteem and overall quality of life [11,12].

In epidemiological terms, temporomandibular disorders (TMDs) likewise show marked geographical variation, with pooled prevalence reported as higher in South America (47%) than in Asia (33%) and Europe (29%) [5]. Complementing these cross-sectional differences, time-trend analyses indicate an increase in TMD prevalence in recent years [13].

Some researchers have suggested that malocclusal abnormalities may be associated with alterations in the musculoskeletal system as a whole, including chronic pain and postural defects [14,15,16,17]. Despite numerous investigations into the relationship between malocclusions, temporomandibular disorders, and musculoskeletal pain, the evidence remains inconsistent. While some studies report significant associations [6,17,18,19], others fail to provide conclusive results [20,21,22]. This inconsistency may result from methodological heterogeneity, varied diagnostic protocols, and limited interdisciplinary approaches. Moreover, recent research indicates that additional factors—including psychological stress, genetic predisposition, and lifestyle—may play equally important roles in the etiology and expression of TMD [23].

The mechanisms that might link malocclusions with TMD and musculoskeletal pain are complex and multifactorial. One explanatory model—the biomechanical theory—proposes that malocclusions may contribute to uneven force distribution during mandibular movements, potentially leading to overload and microtrauma in the TMJ and, over time, degenerative changes. Another model—the neurophysiological theory—suggests that altered occlusion may be associated with abnormal activation of masticatory and perioral muscles, with pain reported along myofascial chains extending to the cervical spine and even more distant structures [24]. Electromyographic studies are consistent with these hypotheses, reporting increased muscular activity in cervical and shoulder regions in patients with malocclusions [25], as well as a higher prevalence of headaches and neck and shoulder pain among this group [26]. Saito et al. [27] further suggested that, once compensatory mechanisms are exceeded, altered alignment of these body regions may occur, potentially increasing overload and adversely affecting the musculoskeletal system as a whole.

Although malocclusions are widely recognized as common dental conditions with functional and psychosocial consequences, their relationship with temporomandibular disorders and pain-related musculoskeletal symptoms remains unclear. This inconsistency underscores the need for further interdisciplinary research that integrates dental and musculoskeletal perspectives.

Therefore, the aim of the present study was to evaluate the coexistence of anteroposterior malocclusions with temporomandibular disorders and pain-related symptoms in the musculoskeletal system in young adults. We hypothesized that anteroposterior malocclusions (Angle Classes II/III) would co-occur with temporomandibular disorders and a higher burden of pain-related musculoskeletal symptoms, particularly within the upper quarter (head, cervical region, shoulder girdle).

## 2. Materials and Methods

### 2.1. Study Design, Recruitment and Participants

This study employed a cross-sectional observational design and was conducted between April 2020 and August 2021 in the Małopolska region, Poland. Participants were recruited primarily from universities in Kraków and a local dental clinic using a convenience sampling approach. Eligibility spanned 18–35 years; however, all enrolled participants were aged 19–35 years [28,29].

Inclusion criteria comprised (1) age 18–35 years, (2) presence of at least 28 permanent teeth, and (3) diagnosis of Angle’s Class I, II, or III malocclusion. Exclusion criteria were: (1) ongoing orthodontic treatment, (2) neurological disorders affecting the central or peripheral nervous system, (3) physician-diagnosed chronic systemic musculoskeletal disorders such as rheumatoid arthritis, ankylosing spondylitis, other spondyloarthritis, generalized osteoarthritis, inflammatory myopathies, fibromyalgia or other chronic widespread pain syndromes, (4) permanent locomotor system injuries or major post-traumatic muscle or tendon injuries leading to persistent structural changes, functional deficit or chronic pain (for example, post-traumatic joint ankylosis, joint replacement, chronic massive rotator cuff tear, spinal fusion), and (5) regular use of analgesic medication or centrally acting muscle relaxants for pain control. All enrolled participants were additionally instructed to refrain from taking any analgesic medication immediately before the clinical examination.

Out of 420 individuals examined, a total of 90 participants (52 women and 38 men) were ultimately enrolled in the study. Consistent with published guidance, this sample size was sufficient to detect large effect sizes with 90% power at α = 0.05 for the planned between-group comparisons across the three occlusal classes [30]. Given the exploratory nature of the study and feasibility constraints in recruiting well-characterized young adults in three occlusal groups, the study was a priori powered to detect large between-class differences in TMD- and pain-related outcomes; smaller or moderate effects may not have been reliably detected. Participants were divided into three groups according to the diagnosed occlusal class. Occlusal class was determined clinically according to Angle’s criteria based on the first permanent molar relationship [31]. Group I included 30 participants (17 women, 13 men) with Angle’s Class I, Group II included 30 participants (16 women, 14 men) with distal occlusion (Angle’s Class II), and Group III included 30 participants (19 women, 11 men) with mesial occlusion (Angle’s Class III) (Table 1).

The study was conducted in accordance with the Declaration of Helsinki, and its protocol was approved by the Bioethics Committee of the District Medical Chamber in Kraków (No. 35/KBL/OIL/2019). All participants provided written informed consent prior to inclusion.

### 2.2. Clinical Procedures and Outcome Measures

The procedure involved a clinical assessment of occlusion and classification according to Angle’s system, followed by a functional examination of the stomatognathic system performed in accordance with the RDC/TMD protocol (Axis I and II). This included standardized evaluation of the temporomandibular joints. Axis II grading referred to the Graded Chronic Pain Scale (GCPS v1.0) embedded within RDC/TMD [32,33]. All examinations were carried out by the same prosthodontist, trained in the application of the RDC/TMD protocol.

Because a clinically implemented, language-adapted DC/TMD protocol was not available in our setting at the time of data collection (April 2020–August 2021), the RDC/TMD (Axis I/II) was used [34]. DC/TMD offers higher diagnostic validity and inter-examiner reliability for pain-related TMD; this is acknowledged as a study limitation. Using this standardized clinical protocol, participants were classified into three primary diagnostic groups: Group I (myofascial pain), Group II (disk displacement), and Group III (arthralgia/arthritis/degenerative joint disease).

A proprietary body-map questionnaire (anterior/posterior views) was developed specifically for this study to capture the presence and intensity of musculoskeletal pain. The layout was adapted from commonly used whole-body pain drawings and body maps for multisite musculoskeletal pain assessment, with the body surface subdivided into predefined anatomical regions (head/face subregions; cervical, thoracic, lumbar and sacroiliac spine; upper and lower limbs) [35]. Head/face subregions included the forehead, temples (left/right), TMJ (left/right), cheeks (left/right), occiput, chin, and vertex. For each region, participants were instructed to mark current pain sites directly on the silhouette and to assign a numerical rating (0–10) next to each marked area (NRS).

The development process comprised several steps. First, the research team defined the set of regions and their graphical boundaries to balance anatomical detail with feasibility of scoring. Second, the preliminary version of the body-map was piloted in a convenience sample of 20 young adults from the same age range as the study population to assess clarity of the instructions, recognizability of regions and overall usability. Participants were asked to complete the map and report any ambiguities or difficulties; minor wording changes and graphic adjustments were made based on this feedback. Third, two independent examiners applied the planned scoring procedure to a subset of completed pilot maps to standardize how marked areas were translated into regional yes/no variables and numeric pain scores; discrepancies were discussed and resolved to produce a final coding manual. In the main study, participants received standardized written and verbal instructions on how to complete the body-map. The primary derived variables were: presence of pain by region (yes/no), NRS intensity by region (0–10 for each marked region) and the total count of painful sites. As the instrument has not undergone formal validation, its use is considered a limitation of the study.

Pain intensity was assessed using the Numerical Rating Scale (NRS), a simple and sensitive tool for the subjective evaluation of pain severity. Study participants indicated the intensity of their pain on a scale from 0 to 10, where 0 represented no pain and 10 denoted the worst imaginable pain. The NRS is characterized by high test–retest reliability and ease of application, which makes it widely used in clinical research and in the assessment of pain intensity [36]. To aid clinical interpretation of NRS (0–10) differences, we prespecified the minimal clinically important difference (MCID) as ~1–2 points or ~30% change from baseline, consistent with established methodological work in chronic pain; in between-group analyses, contrasts ≥1.0 point were interpreted as potentially clinically relevant alongside statistical significance and 95% CIs [37,38].

Reporting followed the STROBE guidelines [39] for observational studies and complied with ICMJE and COPE recommendations.

### 2.3. Statistical Analysis

Analyses were conducted in R, version 4.1.1 (R Core Team, 2021) [40]. Hypothesis testing, effect-size estimation, and multiple-testing correction were performed using the rstatix package [41]. Dunn’s post hoc pairwise comparisons following Kruskal–Wallis tests were obtained using the FSA and dunn.test packages [42,43]. Effect sizes and, where applicable, their 95% confidence intervals (CIs) were computed using the effectsize and DescTools packages [44], as appropriate: η^2^ for Kruskal–Wallis tests; r for Dunn’s post hoc tests (calculated as r = Z/√N and interpreted as small, medium, or large according to Cohen’s framework and dentistry-specific thresholds); Cramér’s V for χ^2^ tests; and Cohen’s d for mean differences in NRS scores and the number of pain sites [30,45]. For selected proportions, absolute percentage-point differences with 95% CIs were also reported to facilitate clinical interpretation. The normality of data distributions was assessed using the Shapiro–Wilk test and visual inspection of Q–Q plots, and the homogeneity of variances was evaluated with Levene’s test (base R/car) [46]. All categorical analyses were performed using χ^2^ tests; expected cell frequencies were inspected to verify the assumptions of the χ^2^ test. To control for multiple testing, Bonferroni correction [47] was applied to all post hoc pairwise comparisons (Dunn’s tests). Bonferroni-adjusted *p*-values (p_adj) were used for inference and, for key post hoc contrasts, are reported together with effect sizes and 95% CIs. Effect-size magnitudes were interpreted as small, medium, or large: η^2^ (≈0.01, 0.06, 0.14), r (≈0.20, 0.40, 0.70), Cramér’s V (≈0.10, 0.30, 0.50), and Cohen’s d (≈0.10, 0.40, 0.90) [30,45]. All analyses were two-tailed, and the significance level was set at *p* < 0.05.

## 3. Results

### 3.1. TMD Prevalence by Occlusal Class

Based on the measurements obtained from the RDC/TMD clinical assessment form, it was possible to diagnose and classify the participants’ symptoms into one of the three groups of temporomandibular disorders: Group I—myofascial pain, Group II—disk displacement, or Group III—arthralgia, arthritis, and degenerative joint disease.

No significant associations were found between occlusal class and the presence of temporomandibular disorders (all *p* > 0.05; Cramér’s V = 0.07–0.20, negligible to small effects), either at the myofascial level or in relation to disk displacement and temporomandibular joint involvement (Table 2). Although these differences were not statistically significant, the prevalence of Group I myofascial disorders was numerically higher in Class III compared with Classes I and II (Class III vs. Class I: Δ = 23.3 percentage points, 95% CI 8.0 to 38.7; Class III vs. Class II: Δ = 13.4 percentage points, 95% CI −3.5 to 30.4).

Axis II analysis revealed significant differences in chronic craniofacial pain grades between occlusal classes (χ^2^(4) = 11.80, *p* = 0.019, Cramér’s V = 0.26, small effect). High-intensity chronic pain (Grade II) was most frequent in Class III (9/30; 30.0%) and less common in Class II (6/30; 20.0%), while it was absent in Class I. In contrast, low-intensity chronic pain (Grade I) was most frequent in Class I (13/30; 43.3%), compared with Class II (7/30; 23.3%) and Class III (10/30; 33.3%).

### 3.2. Pain Outcomes: Craniofacial and Musculoskeletal

Using a proprietary questionnaire containing a human body diagram, participants marked the locations of their current pain symptoms within the musculoskeletal system and the craniofacial region. The analysis included pain location, pain intensity (NRS), and the number of reported areas across individuals with different occlusal types.

The most frequently reported craniofacial pain sites were the occipital region and the right and left temporal regions, with the highest prevalence observed among participants with malocclusions (Table 3). Statistically significant between-class differences were observed only for the left temporal region (*p* = 0.024, Cramér’s V = 0.31, medium effect; Table 3). Pain in this area was most frequently reported by individuals with Class III malocclusion and least frequently by those with normal occlusion (Class III vs. Class I +20.0 pp, 95% CI +1.8 to +38.2; Class III vs. Class II +23.3 pp, 95% CI +6.3 to +40.4). For all other craniofacial sites, no significant associations were found (all *p* ≥ 0.109; Cramér’s V = 0.06–0.25, negligible-to-small effects).

Subsequently, the intensity of pain in the indicated craniofacial regions was assessed using the NRS scale. Participants with Class III malocclusion demonstrated higher mean levels of perceived pain in most of the analyzed sites compared with the other groups (Table 4). Within the Class III group, the highest mean pain levels were recorded in the right and left temporal regions, whereas participants with Class I and Class II malocclusions reported the highest mean pain intensity in the occipital region.

For most sites, Kruskal–Wallis tests did not show statistically significant differences between occlusal classes (all *p* ≥ 0.052; η^2^ = 0.00–0.07, negligible-to-small effects; Table 4). A significant between-class difference was observed only for the left temporal region (H(2) = 8.52, *p* = 0.014, η^2^ = 0.10, medium effect). Post hoc Dunn tests with Bonferroni correction showed higher NRS scores in Class III compared with Class II (p_adj = 0.019, r ≈ 0.29, small-to-medium effect), whereas the difference between Class III and Class I did not remain statistically significant after correction (p_adj = 0.071, r ≈ 0.24, small effect); no difference was found between Class I and Class II (p_adj = 1.000, r ≈ 0.05, negligible). For the left temple, effect estimates for mean NRS differences were as follows: Class III vs. Class I, Δ = 0.90 points (95% CI −0.13 to +1.93; Cohen’s d ≈ 0.44, medium effect) and Class III vs. Class II, Δ = 1.20 points (95% CI +0.31 to +2.09; Cohen’s d ≈ 0.68, medium effect). The magnitude of these differences approaches the lower MCID bound (~1 NRS point) and should be interpreted with caution given the partial loss of statistical significance after correction and the multiple comparisons performed.

The subsequent section of the questionnaire included a human body diagram in anterior and posterior projections. For the purposes of analysis, the body was divided into the following regions: spine, trunk, upper limbs, and lower limbs. The association between occlusal class and spinal pain location was examined using chi-square tests with Cramér’s V as the effect size (Table 5).

A statistically significant difference between occlusal classes was observed only for cervical spine pain (χ^2^(2) = 6.30, *p* = 0.043, Cramér’s V = 0.26, small effect), which was more frequent in individuals with malocclusions (Class II and Class III) than in those with Class I occlusion. For cervical pain occurrence, absolute differences vs. Class I were +23.3 percentage points for Class II (95% CI +4.7 to +42.0) and +23.3 percentage points for Class III (95% CI +4.7 to +42.0). No significant between-class differences were found for thoracic, lumbar or sacroiliac pain (all *p* ≥ 0.094; V = 0.09–0.23, negligible-to-small effects).

Class III malocclusion demonstrated higher mean NRS scores in most spinal regions compared with Class I and Class II (Table 6). The association between occlusal class and spinal pain intensity was examined using Kruskal–Wallis tests with η^2^ as the effect size. A statistically significant between-class difference was found for cervical spine pain (H(2) = 6.95, *p* = 0.031, η^2^ = 0.08, medium effect). However, post hoc Dunn tests with Bonferroni correction did not identify any pairwise comparison that remained statistically significant at α = 0.05 (Class II vs. Class I: p_adj = 0.370, r ≈ 0.16, small effect; Class III vs. Class I: p_adj = 0.232, r ≈ 0.19, small effect; Class III vs. Class II: p_adj = 1.000, r ≈ 0.02, negligible). Nevertheless, mean NRS scores suggested higher cervical pain in malocclusion groups: when Classes II and III were combined, the pooled malocclusion group showed a mean cervical NRS score of 1.43 versus 0.20 in Class I (Δ = 1.23 points, 95% CI 0.38 to 2.08; Cohen’s d ≈ 0.64, medium effect). This difference exceeds the lower MCID threshold (~1 NRS point) and may indicate a clinically relevant burden; however, given that adjusted pairwise comparisons were not statistically significant and multiple pain outcomes were analyzed, this finding should be regarded as exploratory and interpreted with appropriate caution.

Further analyses revealed no significant differences among the study groups with respect to trunk pain. For pain distribution (χ^2^ tests with Cramér’s V), no between-class differences were observed for the right scapular (*p* = 0.318, Cramér’s V ≈ 0.20, small), left scapular (*p* = 0.326, V ≈ 0.21, small), right nape (*p* = 0.487, V ≈ 0.13, small), left nape (*p* = 0.470, V ≈ 0.14, small) or sternal region (*p* = 0.770, V ≈ 0.15, small; all *p* > 0.05). Likewise, NRS pain intensity at these sites did not differ significantly between occlusal classes (right scapular *p* = 0.172, η^2^ ≈ 0.04; left scapular *p* = 0.132, η^2^ ≈ 0.05; right nape *p* = 0.472, η^2^ ≈ 0.02; left nape *p* = 0.358, η^2^ ≈ 0.02; sternum *p* = 0.364, η^2^ ≈ 0.02; all *p* > 0.05), with all η^2^ values indicating small or negligible effects.

For the limbs, no upper-limb pain was reported in any group. In the lower limbs, neither pain occurrence nor NRS intensity showed significant associations with occlusal class. The presence of pain in the right hip (*p* = 1.000, Cramér’s V ≈ 0.00), right knee (*p* = 0.770, V ≈ 0.15, small), left knee (*p* = 0.770, V ≈ 0.15, small) and right calf (*p* = 1.000, V ≈ 0.00; all *p* > 0.05), as well as NRS scores at these sites (right hip *p* = 0.368, η^2^ ≈ 0.02; right knee *p* = 0.351, η^2^ ≈ 0.02; left knee *p* = 0.361, η^2^ ≈ 0.02; right calf *p* = 0.368, η^2^ ≈ 0.02; all *p* > 0.05), were accompanied by negligible-to-small effect sizes, providing no evidence for a strong relationship between occlusal class and trunk or lower-limb pain.

An overall analysis of the relationship between the number of current pain locations in the body and occlusal type, performed with the Kruskal–Wallis test, showed a statistically significant between-class difference (H(2) = 7.57, *p* = 0.023, η^2^ = 0.09, medium effect; Table 7), indicating a meaningful association between occlusal class and the burden of pain. Post hoc Dunn tests with Bonferroni correction demonstrated that this effect was driven primarily by the contrast between Class III and Class I: the Class III group reported, on average, 1.40 more painful sites than Class I (Δ = 1.40 sites, 95% CI 0.39 to 2.41; Cohen’s d ≈ 0.72, medium-to-large effect), and this difference remained statistically significant after adjustment (p_adj ≈ 0.019, r ≈ 0.29, small-to-medium effect). In contrast, the pairwise comparisons involving Class II (Class II vs. Class I and Class III vs. Class II) did not reach statistical significance after Bonferroni correction (all p_adj > 0.05, r ≤ 0.17, small effects).

## 4. Discussion

In line with contemporary medical practice, which increasingly emphasizes a holistic view of the human body, this interdisciplinary study aimed to evaluate the coexistence of malocclusions, musculoskeletal pain, and temporomandibular joint dysfunctions.

No significant association was observed between anteroposterior malocclusions and TMJ disorders (RDC/TMD Axis I). Nevertheless, several statistically and clinically relevant relationships emerged between occlusal type and pain outcomes in the craniofacial region and spine. Participants with Class III malocclusion more frequently reported left temporal pain, and cervical spine pain was more common in malocclusion groups than in Class I, both with small-to-medium effect sizes. For cervical pain intensity, between-class differences reached the prespecified ≈1-point MCID, which may indicate a clinically relevant burden in the malocclusion groups; however, this finding should be interpreted with caution in view of the multiple comparisons performed and only partially consistent post hoc results, whereas temporal NRS differences in Class III approached but did not clearly exceed this threshold. Thus, our data partially confirm the initial hypothesis by demonstrating an association between sagittal malocclusions and the burden and distribution of pain in the upper quarter, but not with TMD prevalence itself, and should be interpreted as associative rather than causal.

Malocclusion is a pathological condition that, as noted by Głowacka [48], can substantially affect the temporomandibular joints, masticatory muscles, and associated tissues. The role of occlusion as an etiological factor in temporomandibular disorders (TMD) has been debated for decades [21,49,50,51]. It is now well established that TMD is multifactorial, with additional determinants such as facial deformities, bruxism, traumatic craniofacial injuries, sex, parafunctions, sleep disturbances, body posture, and psychosocial stress playing important roles [22,24,52,53,54,55,56].

Epidemiological studies conducted by Thilander [57], Alamoudi [58], Celić [59], and Schmitter [60] have demonstrated associations between the occurrence of TMD and various forms of malocclusion, particularly in individuals with crossbite, open bite, deep bite, and Angle’s Class II and III malocclusions. However, most of these investigations were conducted in children, whereas other authors have emphasized that both subjective and clinical TMD symptoms tend to increase with age [61,62,63]. Accordingly, comparisons with our young adult cohort should be viewed as contextual rather than directly extrapolated, given developmental and age-related differences in TMD expression. Recent MRI-based studies have confirmed structural alterations in Class II patients that may predispose them to TMD development [64]. Thilander [57] further highlighted that whether such disturbances progress to TMJ damage depends largely on individual adaptive capacity, personality traits, and susceptibility to stress.

The vast majority of researchers evaluating the coexistence of malocclusions and TMD report no significant associations between these conditions [21,22,65]. When analyzing the anteroposterior relationship of the maxilla and mandible, Rey et al. [65] found no difference in the prevalence of TMD between individuals treated for Class III malocclusion and those with Class I occlusion. These observations are consistent with the results of our study, which also did not demonstrate a correlation between occlusal type (Angle’s Classes I, II, and III) and the occurrence of temporomandibular disorders, including myofascial pain disorders, disk displacements, arthralgia, or degenerative joint disease.

A coherent explanation for the frequent null findings in the occlusion–TMD literature likely combines methodological and biological factors. Methodologically, studies differ in case definitions (RDC/TMD versus DC/TMD), examiner calibration, and sampling frames (adolescents vs. adults; clinical vs. community samples), all of which reduce comparability and statistical power to detect small effects [34,66,67,68]. Biologically, TMD spans a biopsychosocial spectrum in which bruxism, psychosocial distress, and central pain modulation may overshadow relatively small biomechanical inputs from occlusion in many individuals [3,68]. Consistent with this view, recent data from Italian university students by Cannatà et al. [69] have highlighted strong links between pain-related TMD, emotional distress and waking-state oral behaviors in young adults. In addition, the adaptive capacity of the stomatognathic system over time (e.g., neuromuscular adaptation, occlusal wear) can decouple static occlusal morphology from current symptom expression, helping to explain why several high-quality reviews and meta-analyses have concluded that occlusion is not a primary driver of TMD pathophysiology [68]. Importantly, this perspective does not preclude context-dependent contributions of occlusion in selected phenotypes (e.g., pronounced sagittal or transverse discrepancies), but suggests that such effects are unlikely to be predominant at the population level.

The present findings were based on a combination of subjective assessment (patient interview) and objective clinical examination, which helps to reduce the risk of bias. However, certain tendencies were observed. Specifically, the most frequently occurring group of TMD according to the RDC/TMD classification among the study participants was Group I, namely myofascial pain disorders, which were most prevalent in individuals with Class III malocclusion (23.33%). Furthermore, in our study, analysis of Axis II of the RDC/TMD questionnaire revealed the presence of chronic craniofacial pain, with the highest grade and intensity reported in patients with Class III malocclusion and the lowest in those with Class I occlusion. These patterns are descriptive and should be interpreted as reflecting co-occurrence rather than implying a direct causal role of occlusal class.

Manfredini et al. [22], in their review of the literature on the relationship between occlusion and TMD, concluded that although several reports suggest a potential association, there is no basis for the hypothesis that occlusion plays a primary role in the pathophysiology of TMD. The authors emphasize the relevance of the biopsychosocial model in the development of such dysfunctions and highlight the slow adaptation of the stomatognathic system to pre-existing morphological malocclusions [22,70]. From a clinical perspective, this supports the recommendation of conservative, reversible treatment approaches—such as a well-designed occlusal splint, physiotherapy, and pharmacotherapy—as first-line options to control both subjective and objective symptoms of TMD [71].

According to some authors, abnormalities within the stomatognathic system may, through the activity of myofascial chains and the dura mater, influence the tension of adjacent and peripheral structures, gradually generating pain symptoms [72,73,74]. In light of this, the present study aimed to investigate whether individuals with malocclusions experience pain symptoms more frequently and report higher pain intensity in selected body regions. The first area analyzed in the proprietary questionnaire was the head and facial region. Headache is considered one of the primary symptoms of TMD and bruxism [24,75,76,77]. Some authors have suggested that certain malocclusions are significantly associated with an increased prevalence of headaches and partly overlap with malocclusions known to increase the risk of TMD, such as posterior crossbite, anterior open bite, and Angle’s Class II malocclusion [78,79,80]. Lambourne [78] and Al-Shorman and Shdeifat [81] reported that excessive overbite/overjet and posterior crossbite were associated with a higher likelihood of recurrent headaches in children and adolescents without clinical TMD. However, there is a lack of studies directly addressing the association between malocclusions and headache occurrence outside the context of TMD.

In our young adult cohort, we observed a somewhat different tendency. When analyzing pain in the occipital region in relation to the three occlusal classes, symptoms occurred more frequently in individuals with malocclusions and were reported with similar prevalence in those with skeletal Class II and skeletal Class III malocclusions. In contrast, pain in the left temporal region was most prevalent in individuals with Class III malocclusion and least frequent among those with normal occlusion, with a statistically significant association and a medium effect size. At the level of pain intensity, Class III participants also reported higher NRS scores in the temporal regions; however, only the global Kruskal–Wallis test for the left temple reached statistical significance, and Bonferroni-adjusted post hoc comparisons did not consistently confirm pairwise differences between classes. Together with medium effect sizes and NRS differences close to, but not clearly above, the ≈1-point MCID, these results are compatible with a less favorable temporal pain pattern in Class III, but warrant cautious interpretation in view of the partial loss of statistical significance after correction and the number of comparisons performed. Moreover, in the Class III group, pain was more frequently reported in the temporomandibular joints and temporal regions (right and left) compared with individuals with Class II malocclusion and normal occlusion. In turn, participants with Class II malocclusion more often reported pain in the left cheek region compared with the other groups.

The results obtained in our study are consistent with the observations of Wieczorek et al. [82] who used sEMG to assess differences in muscle activity and asymmetry indices across skeletal classes. In individuals with Class III malocclusion, they demonstrated dominance of the anterior temporalis muscles, corresponding anatomically to the temporal region, where in our study pain in this area (left temple) occurred more frequently in Class III participants compared with those in Class I. Conversely, in Class II subjects, the authors found dominance of the masseter muscles, which correspond anatomically to the cheek region. Based on the observations of Wieczorek et al. [82], it may be suggested that increased activity of specific muscles in individuals with Class II and Class III malocclusions, determined by skeletal occlusal type, combined with potential aggravating factors such as emotional stress, anxiety, or the presence of parafunctions, could contribute to heightened tension in these already more engaged muscles and may help to explain the pain patterns observed, although this cannot be verified within our cross-sectional design.

In our homogeneous cohort of university students and recent graduates, tension-pattern phenotypes associated with prolonged study/work posture and screen time—particularly sustained cervical flexion during computer and mobile device use—may interact with Angle class (I–III) to shape site-specific distributions of self-reported pain in the temporal/orofacial and cervical regions [3,83,84]. These occupational and behavioral factors represent important non-occlusal contributors to neck pain that were not directly measured and thus may confound or modify the observed associations. In parallel, trigeminocervical convergence and neuromuscular compensation linked to sagittal discrepancies (e.g., Class III) can increase cervical loading without necessarily meeting RDC/TMD diagnostic thresholds [85]. Within a biopsychosocial framework, bruxism and psychosocial stress may unmask small biomechanical asymmetries in susceptible individuals, whereas more adaptive subjects remain asymptomatic—thereby reconciling our site-specific findings with prior diagnostic-level null results [68]. Beyond these mechanisms, age-related patterns provide an additional explanatory layer. Epidemiological and clinical data show that both TMD signs and broader musculoskeletal pain patterns shift from adolescence into early and middle adulthood, with younger individuals more often displaying fluctuating, predominantly myogenous and localized complaints, and older patients a higher burden of chronic, degenerative and psychosocially complex pain presentations [5,86,87]. Within this developmental continuum, a homogeneous young adult cohort with different sagittal occlusal classes may represent a phase in which neuromuscular adaptation still largely compensates for occlusal discrepancies at the diagnostic TMD level, thereby contributing to the absence of class–TMD associations in our data, while subtle, site-specific differences in craniofacial and cervical pain patterns between classes are already detectable.

Further analyses addressed the occurrence of pain symptoms in different spinal segments and limb regions across the studied occlusal groups. Most of the available literature in this area has focused on the coexistence of temporomandibular disorders with symptoms affecting the head, the spinal column, and even peripheral joints [15,88,89]. Bergamini [90], using surface electromyography, demonstrated that achieving neuromuscular balance of the occlusion reduced mean activation in key postural muscles, including the sternocleidomastoid, spinal extensors, and even the soleus. Valentino et al. [91] likewise employed electromyography to show a functional association between the masticatory system and lower-limb musculature under experimentally induced occlusal disturbances.

Our study identified differences among the examined groups regarding both the location and the overall burden of spinal pain. Cervical spine pain was more frequent in individuals with malocclusions (Classes II–III) than in those with normal occlusion, with small-to-medium effect sizes. Although post hoc pairwise tests for cervical pain intensity did not remain statistically significant after Bonferroni correction, the pooled contrast between malocclusion (Classes II+III) and Class I exceeded the prespecified ≈1-point MCID on the NRS, which may point to a clinically relevant increase in cervical pain burden in the malocclusion groups, but should be regarded as exploratory in light of the multiple comparisons and non-significant adjusted pairwise results. In addition, the overall number of currently painful body sites was higher in Class III than in Class I, with a medium-sized effect, supporting a greater global pain burden in Class III within this cohort, at an associative level rather than implying causality.

Fischer et al. [92] demonstrated a strong association between cranio-mandibular dysfunctions and hip joint mobility limitations and pain, which, according to the authors, may suggest a relationship between these body regions. In the analysis of our own results and malocclusion-related disorders, such observations were not confirmed. According to Fischer, the influence of the stomatognathic system on muscle tension and pain arises from central nervous system pathways transmitting nociceptive information between the TMJ and the rest of the body. Cuccia and Caradonna [15] explained that neural impulses from periodontal proprioceptors can influence shoulder girdle muscles via trigeminal sensory pathways and the accessory nerve, altering the tone of postural muscles. Occlusal imbalance may thus generate aberrant stimuli that could contribute to altered head and neck positioning and pain symptoms. This concept is consistent with our findings, in which pain symptoms in selected body regions were more frequently reported in individuals with malocclusions, particularly those with Class III, most commonly in areas located in close proximity to the stomatognathic system.

Finally, it is important to distinguish statistical from clinical significance. While some contrasts (e.g., cervical NRS in Classes II+III vs. I, number of painful sites in Class III vs. I) were statistically significant and showed medium-sized effects, with mean differences exceeding the prespecified MCID or indicating a clearly higher pain burden, others (e.g., left-temporal NRS in Class III) approached but did not clearly surpass that threshold and were accompanied by small-to-medium effect sizes. Such small effects, even if statistically significant in some settings, are not necessarily clinically meaningful at the individual level and should be interpreted primarily as signals of potential risk patterns rather than as stand-alone treatment indications. Together with multiple-comparison control and the cross-sectional design, this argues for cautious clinical interpretation and replication in longitudinal cohorts [30,37,38].

The cross-sectional design does not permit causal inference; accordingly, our findings should be interpreted as evidence of co-occurrence rather than a causal relationship. Advanced imaging (e.g., MRI) was not performed and could have provided a more granular assessment of TMJ structures. We used RDC/TMD rather than DC/TMD, which may have affected case ascertainment; notably, DC/TMD shows excellent inter-examiner reliability for pain-related TMD (κ ≥ 0.85) [67]. Our proprietary body-map pain questionnaire was not formally validated (e.g., test–retest reliability), introducing potential misclassification of pain location/intensity and bias in effect estimates. We also lacked control for side dominance/laterality (e.g., right/left) when analyzing pain asymmetry (such as left vs. right temple), which could influence side-specific patterns. Objective assessment of bruxism (e.g., nocturnal EMG) was not undertaken and might have masked or attenuated associations between occlusal class, bruxism and pain outcomes. In addition, some confounders—psychosocial stress, parafunctional habits, and lifestyle-related factors—were not fully controlled. Although participants were instructed to abstain from all analgesic medication before the examination, adherence to this recommendation could not be objectively verified, and some residual confounding by intermittent analgesic use cannot be entirely ruled out. Finally, we did not phenotype headaches (e.g., tension-type vs. migraine), which may have attenuated subtype-specific associations.

Despite these limitations, the present work provides unique interdisciplinary evidence by simultaneously examining occlusal type, temporomandibular disorders, and musculoskeletal pain symptoms within one study framework. To our knowledge, only a few studies have addressed these associations in a comparable way, particularly in a homogeneous young adult population. These findings therefore extend current knowledge and underline the need for integrated diagnostic protocols. Future research should build on this foundation by involving larger and more diverse cohorts, employing longitudinal designs, and incorporating standardized diagnostic tools across dental, physiotherapeutic, and psychological domains.

## 5. Conclusions

Although no significant association was observed between occlusal type and TMD occurrence, young adults with sagittal malocclusions—particularly Angle Class III—more frequently reported craniofacial and cervical spine pain and exhibited a higher overall number of painful body sites. These associations were characterized by small-to-medium effect sizes and, for key outcomes such as cervical pain intensity and the total number of painful sites, mean differences that reached or exceeded prespecified MCID thresholds, suggesting a potentially greater pain burden in the malocclusion groups. However, these findings are observational and may be influenced by unmeasured confounding factors; they should therefore be interpreted as patterns of co-occurrence rather than as evidence of a direct causal relationship between occlusal class and pain.

These findings highlight the importance of interdisciplinary assessment in patients with malocclusion, integrating both dental and musculoskeletal evaluation. Future studies with larger and more diverse cohorts, incorporating imaging and longitudinal designs, are warranted to confirm these associations and clarify their prognostic and therapeutic implications.

## Figures and Tables

**Table 1:** Basic demographic characteristics of the study participants.

Group	N	Age [Years], Mean ± SD (Range)	Body Height [cm], Mean ± SD (Range)	Body Weight [kg], Mean ± SD (Range)
I (Angle Class I)	30	22.6 ± 3.1 (19–32)	171.7 ± 9.2 (155–192)	67.3 ± 10.8 (47–90)
II (Angle Class II)	30	23.9 ± 4.2 (19–35)	172.8 ± 7.5 (157–183)	66.9 ± 11.2 (48–91)
III (Angle Class III)	30	22.8 ± 2.9 (20–30)	170.9 ± 8.1 (157–186)	68.2 ± 13.4 (48–100)

N—number of participants; SD—standard deviation; cm—centimeters; kg—kilograms.

**Table 2:** Prevalence of TMD subtypes (RDC/TMD Axis I) across occlusal classes.

Assessed Parameter	Class I N (%)	Class II N (%)	Class III N (%)	Χ^2^ (df)	*p*-Value	Cramér’s V	Magnitude
Group I: Myofascial disorders					0.113	0.20	Small
No disorder	29 (96.7)	26 (86.7)	22 (73.3)	7.46 (4)			
Ia. Myofascial pain	1 (3.3)	4 (13.3)	7 (23.3)				
Ib. Myofascial pain with limited mouth opening	0 (0.0)	0 (0.0)	1 (3.3)				
Group II: Disk displacement (right TMJ)				2.07 (2)	0.355	0.15	Small
Absent	29 (96.7)	30 (100.0)	28 (93.3)				
IIa. Displacement without locking	1 (3.3)	0 (0.0)	2 (6.7)				
IIb. Displacement with locking and restricted range of abduction	0 (0.0)	0 (0.0)	0 (0.0)				
IIc. Displacement with locking and without restricted range of abduction	0 (0.0)	0 (0.0)	0 (0.0)				
Group II: Disk displacement (left TMJ)				0.42 (2)	0.809	0.07	Negligible
Absent	29 (96.7)	28 (93.3)	28 (93.3)				
IIa. Displacement without locking	1 (3.3)	2 (6.7)	2 (6.7)				
IIb. Displacement with locking and restricted range of abduction	0 (0.0)	0 (0.0)	0 (0.0)				
IIc. Displacement with locking and without restricted range of abduction	0 (0.0)	0 (0.0)	0 (0.0)				
Group III: Arthralgia/Arthritis/Arthrosis (right TMJ)				–	–	–	–
Absent	30 (100.0)	30 (100.0)	30 (100.0)				
IIIa. Arthralgia	0 (0.0)	0 (0.0)	0 (0.0)				
IIIb. Arthritis	0 (0.0)	0 (0.0)	0 (0.0)				
IIIc. Arthrosis	0 (0.0)	0 (0.0)	0 (0.0)				
Group III: Arthralgia/Arthritis/Arthrosis (left TMJ)				2.02 (2)	0.364	0.15	Small
Absent	30 (100.0)	29 (96.7)	30 (100.0)				
IIIa. Arthralgia	0 (0.0)	1 (3.3)	0 (0.0)				
IIIb. Arthritis	0 (0.0)	0 (0.0)	0 (0.0)				
IIIc. Arthrosis	0 (0.0)	0 (0.0)	0 (0.0)				

*p*-value—χ^2^ test; effect size—Cramér’s V (negligible < 0.10, small 0.10–0.29, medium 0.30–0.49, large ≥ 0.50); TMJ—temporomandibular joint; N—total in group.

**Table 3:** Association between occlusal type and craniofacial pain.

Pain Location	Pain Occurrence			*p*-Value	Cramér’s *V*	Magnitude
	Class I (N = 30)	Class II (N = 30)	Class III (N = 30)			
Forehead	3 (10.00%)	2 (6.67%)	2 (6.67%)	*p* = 1.000	0.06	negligible
TMJ Right	0 (0.00%)	1 (3.33%)	4 (13.33%)	*p* = 0.122	0.25	small
TMJ Left	2 (6.67%)	1 (3.33%)	3 (10.00%)	*p* = 0.868	0.11	small
Temple Right	3 (10.00%)	2 (6.67%)	8 (26.67%)	*p* = 0.109	0.25	small
Temple Left	2 (6.67%)	1 (3.33%)	8 (26.67%)	*p* = 0.024 *	0.31	medium
Occipital region	4 (13.33%)	8 (26.67%)	8 (26.67%)	*p* = 0.358	0.15	small
Cheek Right	0 (0.00%)	4 (13.33%)	4 (13.33%)	*p* = 0.119	0.22	small
Cheek Left	0 (0.00%)	3 (10.00%)	2 (6.67%)	*p* = 0.363	0.18	small
Chin	1 (3.33%)	0 (0.00%)	1 (3.33%)	*p* = 1.000	0.11	small
Vertex	2 (6.67%)	2 (6.67%)	1 (3.33%)	*p* = 1.000	0.07	negligible

*p*-value—χ^2^ test; effect size—Cramér’s V (negligible < 0.10, small ≥ 0.10, medium ≥ 0.30, large ≥ 0.50); TMJ—temporomandibular joint; N—total in group; * *p* < 0.05.

**Table 4:** Pain intensity (NRS) in craniofacial regions by occlusal class.

Pain Location		Pain Intensity (NRS)			*p*-Value	*η* ^2^	Magnitude
		Class I (N = 30)	Class II (N = 30)	Class III (N = 30)			
Forehead	Mean ± SD	0.4 ± 1.3	0.4 ± 1.61	0.2 ± 0.92	*p* = 0.866	0.00	negligible
	Median	0	0	0			
	Quartiles	0-0	0-0	0-0			
TMJ Right	Mean ± SD	0 ± 0	0.1 ± 0.55	0.7 ± 1.93	*p* = 0.062	0.06	medium
	Median	0	0	0			
	Quartiles	0-0	0-0	0-0			
TMJ Left	Mean ± SD	0.13 ± 0.57	0.1 ± 0.55	0.53 ± 1.76	*p* = 0.561	0.01	small
	Median	0	0	0			
	Quartiles	0-0	0-0	0-0			
Temple Right	Mean ± SD	0.4 ± 1.3	0.3 ± 1.15	1.33 ± 2.35	*p* = 0.052	0.07	medium
	Median	0	0	0			
	Quartiles	0-0	0-0	0-2.25			
Temple Left	Mean ± SD	0.43 ± 1.65	0.13 ± 0.73	1.33 ± 2.37	*p* = 0.014 * Cl.III > Cl.I, Cl.II	0.10	medium
	Median	0	0	0			
	Quartiles	0-0	0-0	0-2.25			
Occipital region	Mean ± SD	0.47 ± 1.22	1.23 ± 2.18	1.1 ± 2.07	*p* = 0.292	0.03	small
	Median	0	0	0			
	Quartiles	0-0	0-2.25	0-0.75			
Cheek Right	Mean ± SD	0 ± 0	0.5 ± 1.33	0.8 ± 2.09	*p* = 0.113	0.05	small
	Median	0	0	0			
	Quartiles	0-0	0-0	0-0			
Cheek Left	Mean ± SD	0 ± 0	0.33 ± 1.03	0.37 ± 1.4	*p* = 0.240	0.03	small
	Median	0	0	0			
	Quartiles	0-0	0-0	0-0			
Chin	Mean ± SD	0.07 ± 0.37	0 ± 0	0.13 ± 0.73	*p* = 0.603	0.01	small
	Median	0	0	0			
	Quartiles	0-0	0-0	0-0			
Vertex	Mean ± SD	0.2 ± 0.76	0.43 ± 1.7	0.1 ± 0.55	*p* = 0.798	0.01	small
	Median	0	0	0			
	Quartiles	0-0	0-0	0-0			

*p*-value—Kruskal–Wallis test; effect size—η^2^ (negligible <0.01, small ≈ 0.01, medium ≈ 0.06, large ≈ 0.14); * *p* < 0.05; TMJ—temporomandibular joint; N—total in group; NRS—Numerical Rating Scale.

**Table 5:** Type of occlusion and the occurrence of pain symptoms in different regions of the spine.

Pain Location	Pain Occurrence			*p*-Value	Cramér’s *V*	Magnitude
	Class I (N = 30)	Class II (N = 30)	Class III (N = 30)			
C	2 (6.67%)	9 (30.00%)	9 (30.00%)	*p* = 0.043 *	0.26	small
Th	3 (10.00%)	2 (6.67%)	4 (13.33%)	*p* = 0.690	0.09	negligible
L	4 (13.33%)	2 (6.67%)	8 (26.67%)	*p* = 0.094	0.23	small
Sacroiliac joints	0 (0.00%)	0 (0.00%)	1 (3.33%)	*p* = 0.364	0.15	small

*p*-value—χ^2^ test; effect size—Cramér’s V (negligible < 0.10, small ≥ 0.10, medium ≥ 0.30, large ≥ 0.50); C—cervical; Th—thoracic; L—lumbar; * *p* < 0.05; N—total in group.

**Table 6:** Type of occlusion and pain intensity in different regions of the spine.

Pain Location		Pain Intensity (NRS)			*p*-Value	η^2^	Magnitude
		Class I (N = 30)	Class II (N = 30)	Class III (N = 30)			
C	Mean ± SD	0.2 ± 0.81	1.27 ± 2.07	1.6 ± 2.54	*p* = 0.031 *	0.08	medium
	Median	0	0	0			
	Quartiles	0-0	0-3	0-4			
Th	Mean ± SD	0.43 ± 1.38	0.27 ± 1.01	0.77 ± 2.03	*p* = 0.639	0.01	small
	Median	0	0	0			
	Quartiles	0-0	0-0	0-0			
L	Mean ± SD	0.53 ± 1.41	0.27 ± 1.05	1.27 ± 2.23	*p* = 0.077	0.06	medium
	Median	0	0	0			
	Quartiles	0-0	0-0	0-2.25			
Sacroiliac joints	Mean ± SD	0 ± 0	0 ± 0	0.23 ± 1.28	*p* = 0.368	0.02	small
	Median	0	0	0			
	Quartiles	0-0	0-0	0-0			

*p*-value—Kruskal–Wallis test; effect size—η^2^ (small ≈ 0.01, medium ≈ 0.06, large ≈ 0.14); * *p* < 0.05; C—cervical spine; Th—thoracic spine; L—lumbar spine; N—total in group; NRS—Numerical Rating Scale; Cl.—Class.

**Table 7:** Number of pain sites identified in the body across different occlusal classes.

Pain Site Count	Occlusal Type			*p*-Value	η^2^	Magnitude
	Class I (N = 30)	Class II (N = 30)	Class III (N = 30)			
Mean ± SD	1.33 ± 1.58	1.80 ± 1.81	2.73 ± 2.24	*p* = 0.023 *	0.09	medium
Median	1	2	2			
Quartiles	0-2	0.25-2	1-4	Cl.III > Cl.I		

*p*-value—Kruskal–Wallis test; effect size—η^2^ (small ≈ 0.01, medium ≈ 0.06, large ≈ 0.14); * *p* < 0.05; Cl.—Class.

## Data Availability

Data are contained within the article.

## Abbreviations

The following abbreviations are used in this manuscript:

TMD	Temporomandibular disorders
TMJ	Temporomandibular joint
ENT	Otolaryngology
RDC/TMD	Research Diagnostic Criteria for Temporomandibular Disorders
DC/TMD	Diagnostic Criteria for Temporomandibular Disorders
GCPS v1.0	Graded Chronic Pain Scale, version 1.0.
NRS	Numerical Rating Scale
MCID	Minimal Clinically Important Difference
CI	Confidence interval
STROBE	Strengthening the Reporting of Observational Studies in Epidemiology
ICMJE	International Committee of Medical Journal Editors
COPE	Committee on Publication Ethics
sEMG	Surface electromyography
MRI	Magnetic resonance imaging
pp	Percentage points
N	Sample size
C/Th/L	Cervical/Thoracic/Lumbar spine segments
